# Clustering compositional data using Dirichlet mixture model

**DOI:** 10.1371/journal.pone.0268438

**Published:** 2022-05-18

**Authors:** Samyajoy Pal, Christian Heumann

**Affiliations:** Department of Statistics, LMU Munich, Munich, Bayern, Germany; IIT Madras, INDIA

## Abstract

A model-based clustering method for compositional data is explored in this article. Most methods for compositional data analysis require some kind of transformation. The proposed method builds a mixture model using Dirichlet distribution which works with the unit sum constraint. The mixture model uses a hard EM algorithm with some modification to overcome the problem of fast convergence with empty clusters. This work includes a rigorous simulation study to evaluate the performance of the proposed method over varied dimensions, number of clusters, and overlap. The performance of the model is also compared with other popular clustering algorithms often used for compositional data analysis (e.g. KMeans, Gaussian mixture model (GMM) Gaussian Mixture Model with Hard EM (Hard GMM), partition around medoids (PAM), Clustering Large Applications based on Randomized Search (CLARANS), Density-Based Spatial Clustering of Applications with Noise (DBSCAN) etc.) for simulated data as well as two real data problems coming from the business and marketing domain and physical science domain, respectively. The study has shown promising results exploiting different distributional patterns of compositional data.

## Introduction

In statistics, compositional data are quantitative descriptions of the parts of some whole, which means that it consists of relative information [1]. Mathematically, compositional data follows the Aitchison geometry on the simplex [2]. Measurements including probabilities, proportions, percentages, and ppm can all be thought of as compositional data. In general, compositional data is written as,

$$\begin{array}{c} S^{D}=\left\{x=\left[x_{1},x_{2},...,x_{D}\right]\in R^{D}|x_{i}>0,i=1,2,...,D;\sum_{i=1}^{D}x_{i}=c\right\} \end{array}$$ (1)

In other words, compositional data is a *D* dimensional real vector, *x* = [*x*_1_, *x*_2_, …, *x*_*D*_] of positive components on $R^{D}$ such that the sum of all components is *c*. Often, we observe the sum of all components to be 1; if not, all the components are divided by the sum of all components, such that $\sum_{i=1}^{D}x_{i}=1$. Analysis of such data is widely used in the fields of geochemistry [3, 4], biology [5–7], ecology [8, 9], finance and business studies [10–12], etc. But it has emerged in the literature long before. [13] identified the problem of ‘spurious correlation’ between ratios of variables and [14] later extended the work and showed that some of the correlations between components of the composition must be negative because of the unit sum constraint. Many transformations have been proposed over the years (e.g. log transformation [15], log ratio transformation [16]) to overcome the unit sum constraint, but still it is argued when it comes to choosing the best transformation [17].

Another issue with compositional data refers to the dealing with zero values as both ratios as well as logarithms are operations that require non-zero elements in the data matrix. Many researchers have tried different approaches to deal with zero values (see [18–21]), but it remains as an open problem even today; mostly because, zero values occur in compositional data for different reasons. Often, the “zero problem” is linked with the missing data problem. Missing data are generally classified into three categories [22], namely: missing completely at random (MCAR), missing at random (MAR) and not missing at random (NMAR). In compositional data analysis, the rounded zeros are considered a NMAR case, where data cannot be observed because their values are below a known value *ϵ*. Zero values can also occur when the count of an element is zero (known as count zero) and when zero signifies some property or relevant information (known as essential zeros). For our study of compositional data in cluster analysis, we have encountered round zeros and we have used a method proposed by [20], where we replace the zeros with a small quantity and adjust others in a multiplicative way which does not affect the covariance structure of the data. The adjusted values *xr*_*ij*_ can be written as

$$\begin{array}{c} xr_{ij}=\{\begin{array}{cc} \delta_{ij} & \text{if}\;\;x_{ij}=0\\ x_{ij}(1-\frac{\sum_{k|x_{ik}=0}\delta_{ik}}{c_{i}}) & \text{if}\;\;x_{ij}>0 \end{array} \end{array}$$ (2)

where *c*_*i*_ is usually the sum constraint. For row *i* and component *j*, the above adjustment in Eq 2 replaces the component *x*_*ij*_ by a very small quantity *δ*_*ij*_ if *x*_*ij*_ = 0, else it multiplies a term $\left(1-\frac{\sum_{k|x_{ik}=0}\delta_{ik}}{c_{i}}\right)$ with *x*_*ij*_ to maintain the unit sum constraint. Here, *x*_*ik*_’s are the zero components in row *i*. The multiplicative term is a fraction by which the non-zero terms to be reduced in order to accommodate the added values of *δ*_*ik*_’s and keep the sum of rows fixed at *c*_*i*_.

For clustering compositional data there exists many methods in the literature [23, 24]. We generally see two kinds of approaches, namely; model based methods, e.g. mixture models [25] and methods based on dissimilarity distances (e.g. hierarchical clustering [26], KMeans [27]. But most of the time researchers go for Gaussian mixture model or KMeans for clustering purposes [28].

For estimating the parameters of mixture models, the EM algorithm [29–31] is widely used. In many applications of mixture models, e.g. in image matching [32], and audio and video scene analysis [33], the EM algorithm is being used regularly. But the EM algorithm is often not very convenient to apply for other than normal distributions, because it needs to be modified and adapted for each case. Sometimes, updating the parameters in the M step becomes impossible for some distributions [34].

The main objectives of our study are to

- develop a clustering method without the need of transformation of compositional data,
- build a mixture model with distribution other than normal,
- evaluate the performance of the method in different situations (different dimensions, different number of clusters and varied overlap).

We are going to propose a model based clustering method without transformation of compositional data. We have used a Hard EM [35] with some modifications, to build mixture models using Dirichlet distribution. For that purpose we need some point estimates of the parent distributions. It is very convenient as it works with both, likelihood based and Bayesian estimates. But the problem with hard assignment of cluster is that it ignores cluster membership probabilities of less probable clusters. As a result, often the algorithm converges too quickly with one or more clusters being empty. In our study we have also proposed a way to deal with that problem. We have done rigorous simulation study to evaluate the performance of the proposed method over varying dimension, number of clusters and overlap. We have also used two real dataset from business and physical science domain to illustrate the method.

## Methodology

Let *X*_1_, *X*_2_, …, *X*_*N*_ denote a random sample of size *N*, where *X*_*i*_ is a *p* dimensional random vector with probability density function *f*(*x*_*i*_) on $R^{p}$. We can write $X={\left(X_{1}^{T},\ldots ,X_{N}^{T}\right)}^{T}$, where the superscript *T* denotes vector transpose. Note that the entire sample is represented by *X*, i.e. *X* is a *N*—tuple of points in $R^{p}$ or an *N* × *p*-matrix. $x={\left(x_{1}^{T},\ldots ,x_{N}^{T}\right)}^{T}$ denotes an observed random sample where *x*_*i*_ is the observed value of the random vector *X*_*i*_.

The density of a mixture model with k components for one observation *x*_*i*_ is given by the mixture density

$$\begin{array}{c} p\left(x_{i}\right)=\sum_{j=1}^{k}\pi_{j}f_{j}\left(x_{i}|\alpha_{j}\right)\;, \end{array}$$ (3)

where *π* = (*π*_1_, …, *π*_*k*_) contains the corresponding mixture proportions with $\sum_{i=1}^{k}\pi_{i}=1$, 0 ≤ *π*_*i*_ ≤ 1. *f*_*j*_(*x*_*i*_|*α*_*j*_) is the density component of mixture *j* and *α*_*j*_, *j* = 1, 2, …, *k*, are vectors of component specific parameters for each density. Then *α* = (*α*_1_, …, *α*_*k*_) denotes the vector of all parameters of the model. The log likelihood of the model for a sample of size *N* is then given by

$$\begin{array}{c} \text{log}p\left(x_{1},\ldots ,x_{N}|\alpha ,\pi \right)=\sum_{i=1}^{N}\text{log}[\sum_{j=1}^{k}\pi_{j}f_{j}\left(x_{i}|\alpha_{j}\right)]\;. \end{array}$$ (4)

The parameters can be estimated using the EM algorithm with some modifications. For that purpose, let us introduce latent variables *Z*_*i*_, which are categorical variables taking on values 1, …, *k* with probabilities *π*_1_, …, *π*_*k*_ such that *Pr*(*X*_*i*_|*Z*_*i*_ = *j*) = *f*_*j*_(*x*_*i*_), *j* = 1, …, *k*. Further, probabilities *γ*_*ij*_ are introduced (conditional on the observed data *X* = *x* and the parameters *α*):

$$\begin{array}{c} \gamma_{ij}\left(x_{i}\right)=Pr\left(Z_{i}=j|X=x,\alpha \right)=\frac{\pi_{j}f_{j}\left(x_{i}|\alpha_{j}\right)}{\sum_{j=1}^{k}\pi_{j}f_{j}\left(x_{i}|\alpha_{j}\right)}\;. \end{array}$$ (5)

Eq 5 can be seen as a cluster membership probability of data point *i* for cluster *j*. For an EM algorithm, we try to optimize the function

$$\begin{array}{c} Q\left(\alpha ,\alpha^{t-1}\right)=E[\sum_{i=1}^{N}\text{log}\left(p\left(x_{i},z_{i}|\alpha \right)\right)|x,\alpha^{t-1}]\;, \end{array}$$ (6)

where *t* is the current iteration number. It is nothing but the expected complete data log likelihood. It can also be shown that (see [36]),

$$\begin{array}{c} Q\left(\alpha ,\alpha^{t-1}\right)=\sum_{i=1}^{N}\sum_{j=1}^{k}\gamma_{ij}\text{log}\pi_{j}+\sum_{i=1}^{N}\sum_{j=1}^{k}\gamma_{ij}\text{log}f_{j}\left(x_{i}|\alpha_{j}\right)\;, \end{array}$$ (7)

where the expected complete data log likelihood is expressed as sum of two parts. At the M step, we optimize *Q* with respect to *π* and *α*. *π*_*j*_ is estimated in the usual way by $\frac{N_{j}}{N}$, where, $N_{j}=\sum_{i=1}^{N}\gamma_{ij}$ and for estimating *α*, we look at the part in *Q* (Eq 7) which depends on *α*, which is given by,

$$\begin{array}{c} l\left(\alpha \right)=\sum_{i=1}^{N}\sum_{j=1}^{k}\gamma_{ij}\text{log}f_{j}\left(x_{i}|\alpha_{j}\right) \end{array}$$ (8)

Now, we choose *α*_*j*_ such that $\alpha_{j}^{t}=\underset{\alpha_{j}}{\text{argmax}}l\left(\alpha_{j}\right)$, which is obtained by the process of assigning data points to respective clusters, given by $\underset{j}{\text{argmax}}\gamma_{ij}$, and estimate *α*_*j*_ by some estimation method based on the assigned observations to that cluster. It can be seen as a Bayesian concept (although not strictly Bayesian) for learning where Eq 5 provides the cluster membership probability. The idea of choosing the cluster based on maximum probability is the same as choosing the MAP estimate, the mode of the distribution of *Pr*(*Z*_*i*_ = *j*|*X*, *α*).

To run the algorithm, at first, some trial values of the distribution parameters *α* and mixture proportions *π* are initialized. Then the initial value of the log likelihood is evaluated. For different distributions, different techniques can be used to choose suitable initial values. For example, in the case of a GMM, the centroids of KMeans can be used as initial values of *μ* and the empirical covariance matrix of each cluster can be taken as an initial value of Σ_*j*_. On the other hand, for a Dirichlet Mixture Model, centroids of KMeans can be multiplied with a scalar *c* (for our study we have used *c* = 60) to get the initial values of the *α* parameters. Please recall that the mean vector of a Dirichlet distribution consists of the ratios of *α* parameters and the sum of all *α* parameters. Here, the scalar *c* acts as the sum of *α* parameter values. The initial values of *π* can be obtained by generating a random number from a Dirichlet (1,1,1,…,1) distribution. The empirical ratios of the number of cluster members in the KMeans algorithm and total observations can also be used as the initial values of *π*. For our study, we have used the KMeans initialization technique mentioned above for all our experiments.

At the E step, the values of the probabilities *γ*_*ij*_ are evaluated using the current parameter values. For an usual EM algorithm (e.g. in a GMM), at the M step, a weighted mean and a weighted covariance matrix are calculated using the *γ*_*ij*_ values. But for other distributions, where the model parameters are not mean and (co)variance, this technique can not be used. So, for different distributions, different techniques needs to be used. And also, for such Hard EM, sometimes the algorithm converges with one or more clusters being empty. Hence, one might have to force the algorithm to re-iterate if one or more clusters are found to be empty at each M step. To introduce a flexible, yet convenient solution, we propose a different technique in our algorithm, where at the M step each data point is assigned to a cluster depending on the probability of that data point belonging to each cluster. That cluster is assigned for which the probability is maximum. Now, if one or more clusters are found empty then the initial value of the parameter *α*_*j*_ for empty cluster *j* is used. And for the non empty clusters, point estimates of the parameters of each parent distributions are obtained using only the data points available in each cluster. For faster convergence and convenience, maximum likelihood estimates can usually be recommended. The mixture component probabilities *π*_*j*_ are estimated as mentioned above by $\frac{N_{j}}{N}$. The newly set of estimated values of the parameters is then used as an update over the previous one. After this step, the log likelihood is evaluated again using the updated parameter values. The process is then continued until convergence. The convergence properties of this algorithm follow the properties of the usual EM algorithm, which has been explained in detail by [37, 38].

**Algorithm 1**: Clustering algorithm for mixture of Dirichlet distributions with provision for empty clusters (Hard DMM 1)

Replace zero values in the data, if any, using Eq 2;

Initialize the model parameters, *α* and *π*. Evaluate the initial value of the log likelihood from Eq 4;

**while** *log likelihood difference* ≥ *ϵ* **do**

 Evaluate *γ*_*ij*_ from Eq 5, using the parameter values and data;

 $\pi_{j}^{new}=\frac{N_{j}}{N}$, where, $N_{j}=\sum_{i=1}^{N}\gamma_{ij}$;

 **for** *i in 1 to N* **do**

  $\text{cluster}=\underset{j}{\text{argmax}}\gamma_{ij}$;

  Assign data point *x*_*i*_ to cluster *z*_*i*_;

 **end**

 **for** *j in 1 to k* **do**

  **if** *cluster j is empty* **then**

   Use initial values of *α*_*j*_ as an update;

  **else**

   $\alpha_{j}^{new}=\alpha_{j}^{MLE}$;

  **end**

 **end**

 Re-evaluate log likelihood using the new estimates of the parameters.

**end**

For our experiments, we have used 0.0001 as the value of *ϵ* in Algorithm 1.

For clustering compositional data, a Dirichlet Mixture Model can be used. The Dirichlet density component *j* is given by

$$\begin{array}{ccc} f_{j}\left(x_{i}\right) & = & \frac{\Gamma (\sum_{m=1}^{p}\alpha_{jm})}{\prod_{m=1}^{p}\Gamma \left(\alpha_{jm}\right)}\prod_{m=1}^{p}x_{im}^{\alpha_{jm}-1},\\ & & where\sum_{m=1}^{p}x_{im}=1,x_{im}’\text{s}>0\;,\alpha_{jm}’\text{s}>0\;. \end{array}$$ (9)

If we make a finite mixture with *k* components, the model is given by Eq 3 and subsequently, the log likelihood is given by Eq 4.

The model parameters, can be easily estimated using our generalized approach. For that we need a good point estimate of the parameters of a Dirichlet distribution to be used in the M step of our algorithm. [39] has discussed a way to find out the maximum likelihood estimates of a Dirichlet distribution, where he proposed to perform a fixed point iteration, given an initial value of the *α* parameters. The equation is given by

$$\begin{array}{c} \Psi \left(\alpha_{jm}^{new}\right)=\Psi \left(\sum_{m=1}^{p}\alpha_{jm}^{old}\right)+\frac{1}{N_{j}}\sum_{i=1}^{N_{j}}\text{log}\left(x_{im}\right) \end{array}$$ (10)

At each iteration, for an old value of the parameter $\alpha_{jm}^{old}$, a new value $\alpha_{jm}^{new}$ is obtained. This iteration in the algorithm requires inverting Ψ, which is a digamma function. A suitable initial value and inversion algorithm is also discussed by [39].

### A special provision of Bayesian estimates for clusters with fewer data points

It is possible to add a further step in algorithm 1 to consider the case when there are very few data points in a cluster due to hard assignment. In this situation, Bayesian estimates can be very useful as they can use some prior information about the model parameters. Also, for fewer data points, maximum likelihood estimates are known to be less accurate. However, Bayesian estimation of Dirichlet parameters is tricky due to several reasons. Even though the Dirichlet distribution has a conjugate prior for being a member of the exponential family, the posterior distribution is difficult to use in practical problems and not analytically tractable. Few authors have proposed some approximation to the posterior distribution of Dirichlet parameters (e.g. [40] have used multivariate Gaussian distribution), but no method seems to yield satisfactory results. Also, using some Markov Chain Monte Carlo (MCMC) algorithm at each iteration step of the clustering algorithm makes it too time-consuming, which is not practically feasible. Considering all these challenges, we are going to propose a suitable solution that can be adopted in our clustering algorithm.

Let us recall that, if (*X*_1_, *X*_2_, …, *X*_*p*_) follows a Dirichlet distribution, with parameters (*α*_1_, *α*_2_, …, *α*_*p*_) then the marginal distribution of *X*_*i*_ follows a Beta distribution with parameters $\left(\alpha_{i},\sum_{j=1}^{p}\left(\alpha_{j}-\alpha_{i}\right)\right)$. Now, if we choose the prior distribution of *α*_*i*_ as *Gamma* (*a*, *b*), then under certain assumptions, the posterior distribution of *α*_*i*_ can be obtained in closed form. It can be shown that (see [41]), posterior distribution of *α*_*i*_ follows a Gamma distribution with parameters (*a* + *n*) and $\frac{1}{b-\sum_{i=1}^{n}log\;x_{i}}$, where *n* is the sample size.

Thus, for our clustering problem, the Bayesian estimates of *α*_*jm*_, *m* = 1, 2, …*p* for cluster *j* can be obtained by the posterior mean, which is given by,

$$\begin{array}{c} \alpha_{jm}^{Bayes}=E\left(\alpha_{jm}\right)=\frac{a+N_{j}}{b-\sum_{i=1}^{N_{j}}log\;x_{im}} \end{array}$$ (11)

For our experiment, we have chosen the values of *a* and *b* to be 1. The extended algorithm with Bayesian estimates for clusters with fewer data points (Hard DMM 2) is explained below.

**Algorithm 2**: Clustering algorithm for mixture of Dirichlet distributions with special provision for clusters with fewer data points (Hard DMM 2)

Replace zero values in the data, if any, using Eq 2;

Initialize the model parameters, *α* and *π*. Evaluate the initial value of the log likelihood from Eq 4;

**while** *log likelihood difference* ≥ *ϵ* **do**

 Evaluate *γ*_*ij*_ from Eq 5, using the parameter values and data;

 $\pi_{j}^{new}=\frac{N_{j}}{N}$, where, $N_{j}=\sum_{i=1}^{N}\gamma_{ij}$;

 **for** *i in 1 to N* **do**

  $\text{cluster}=\underset{j}{\text{argmax}}\gamma_{ij}$;

  Assign data point *x*_*i*_ to cluster *z*_*i*_;

 **end**

 **for** *j in 1 to k* **do**

  **if** *cluster j is empty* **then**

   Use initial values of *α*_*j*_ as an update;

  **else**

   *if* *number of data points in cluster j* ≥ 30 **then**

    $\alpha_{j}^{new}=\alpha_{j}^{Bayes}$;

   **else**

    $\alpha_{j}^{new}=\alpha_{j}^{MLE}$;

   **end**

  **end**

 **end**

 Re-evaluate log likelihood using the new estimates of the parameters.

**end**

## Simulation study

### Comparison with other clustering algorithms

We have done simulation study to check the efficiency of the proposed technique. For a Hard DMM, algorithm 1 and algorithm 2 can be used without any alteration. The objective of our simulation study is to compare the performance of Hard DMM 1 and Hard DMM 2 with other popular clustering algorithms which researchers often use for clustering compositional data. For our study we have considered hierarchical agglomerative clustering with linkage criteria ward, single, average and complete respectively [42]. We have also used partition around medoids (PAM) [43], Clustering Large Applications based on Randomized Search (CLARANS) [44], Fuzzy CMean [45], Kmeans, Gaussian Mixture Model (GMM), Gaussian Mixture Model with hard EM (Hard GMM), spectral clustering [46] and DBSCAN [47] for comparison. We have checked three measures to evaluate the performance.

- Accuracy: The total accurate classifications divided by number of observations.
- Precision: True positives divided by sum of true positives and false positives.
- Recall: True positives divided by the sum of true positives and false negatives.

A detail description of all the measures can be found in [48].

In this section, we have generated data under two schemes.

- **Scheme 1**: 500 random samples from Dirichlet(30,20,10), 100 random samples from Dirichlet (10,20,30) and 300 random samples from Dirichlet (15,15,15).
- **Scheme 2**: 500 random samples from Dirichlet(10,10,3), 100 random samples from Dirichlet (10,20,50), 300 random samples from Dirichlet (15,15,15) and 400 random samples from Dirichlet(0.2,0.5,3)

The data has been generated in python programming language using numpy library [49]. The algorithms of Hard DMM 1, Hard DMM 2 and Hard GMM are also written in python programming language. All the hierarchical clustering algorithms, PAM, KMeans, GMM spectral clustering and DBSCAN algorithm are available in python from scikit-learn, a machine learning library in python [50]. The algorithm for Fuzzy CMean is available in scikit-fuzzy python library [51] and CLARAN is available in PyClustering library [52].

**Fig 1:**
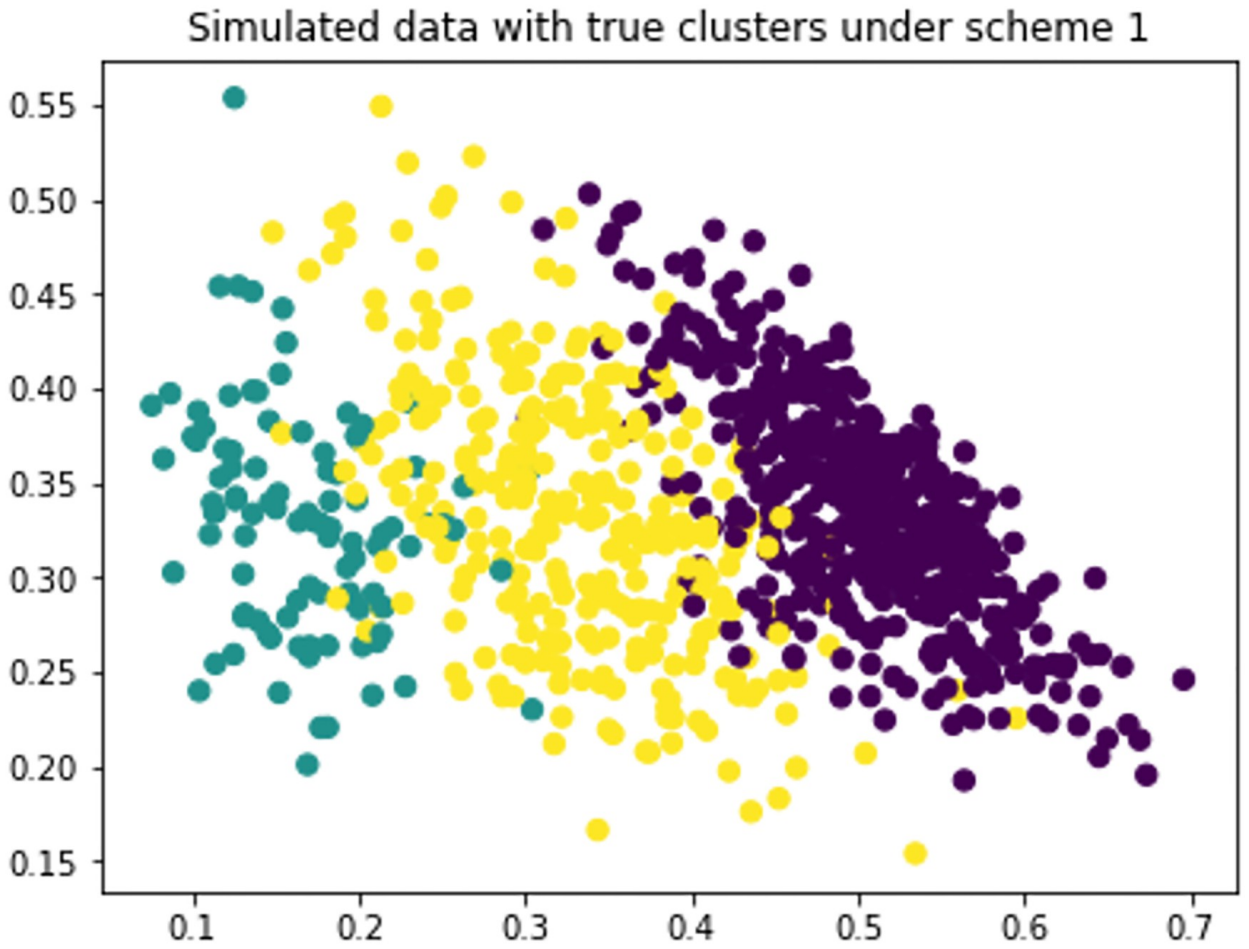
Simulated data generated under scheme 1 with true clusters.

**Fig 2:**
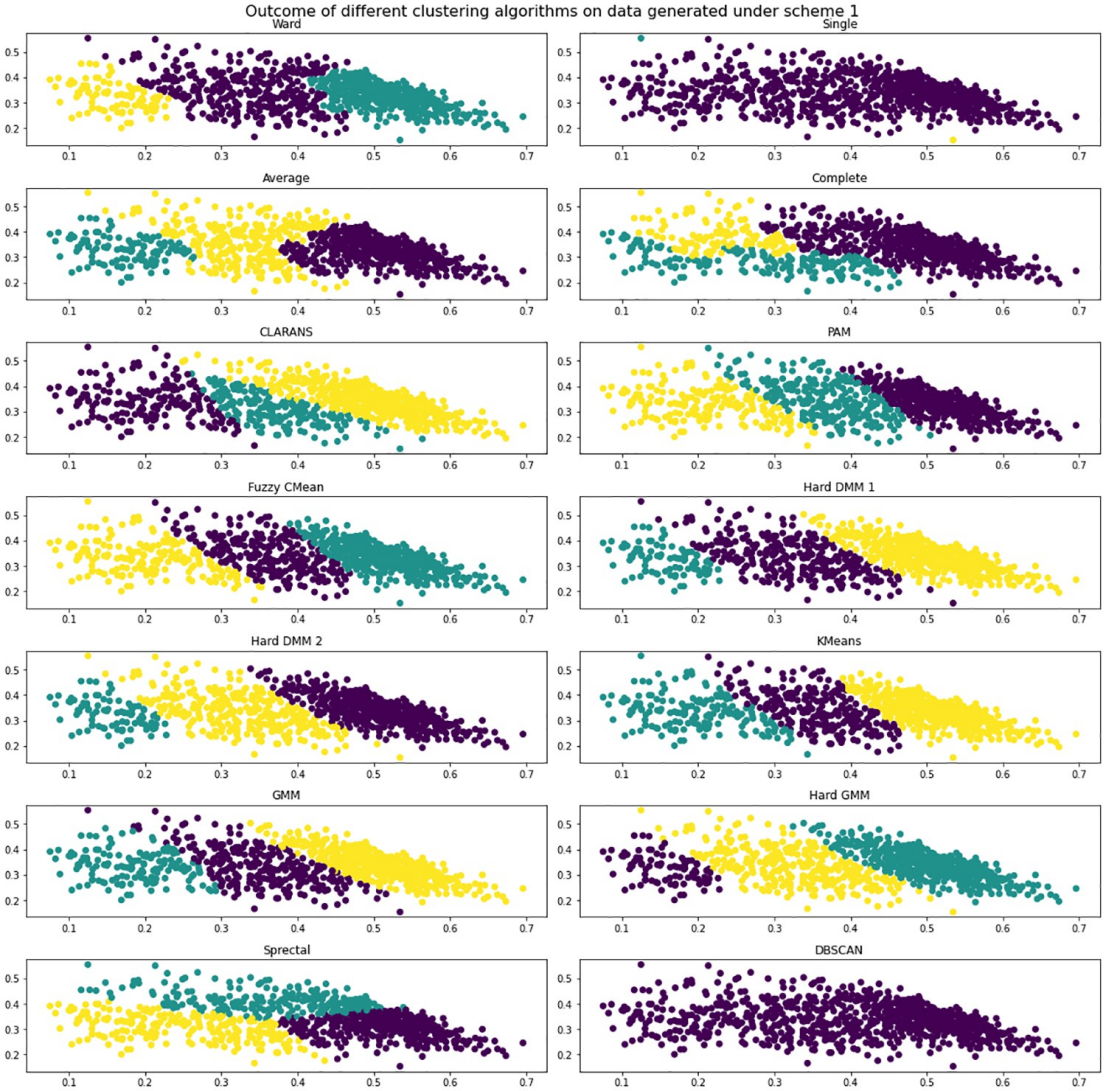
Outcome of different clustering algorithms on simulated data generated under scheme 1.

**Fig 3:**
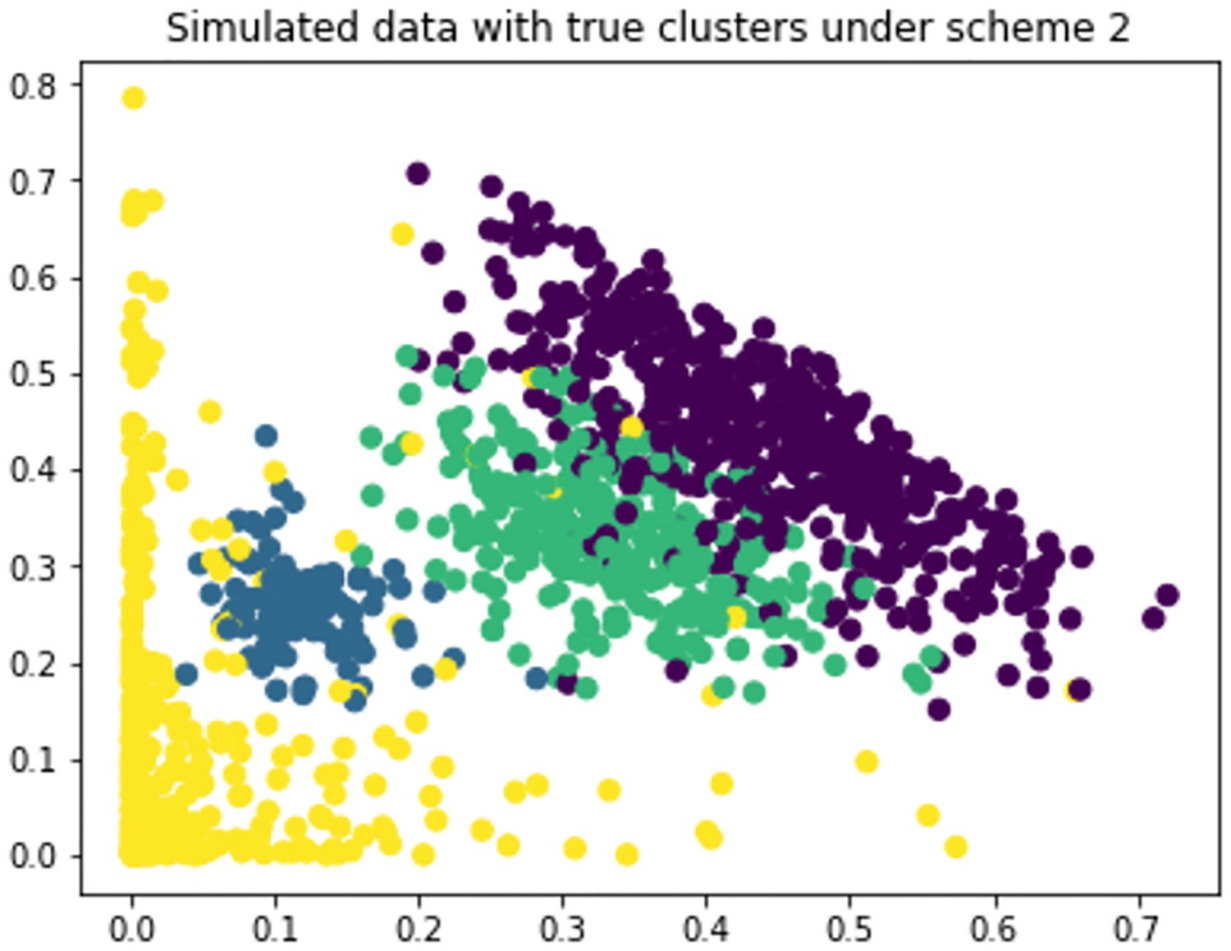
Simulated data generated under scheme 2 with true clusters.

**Fig 4:**
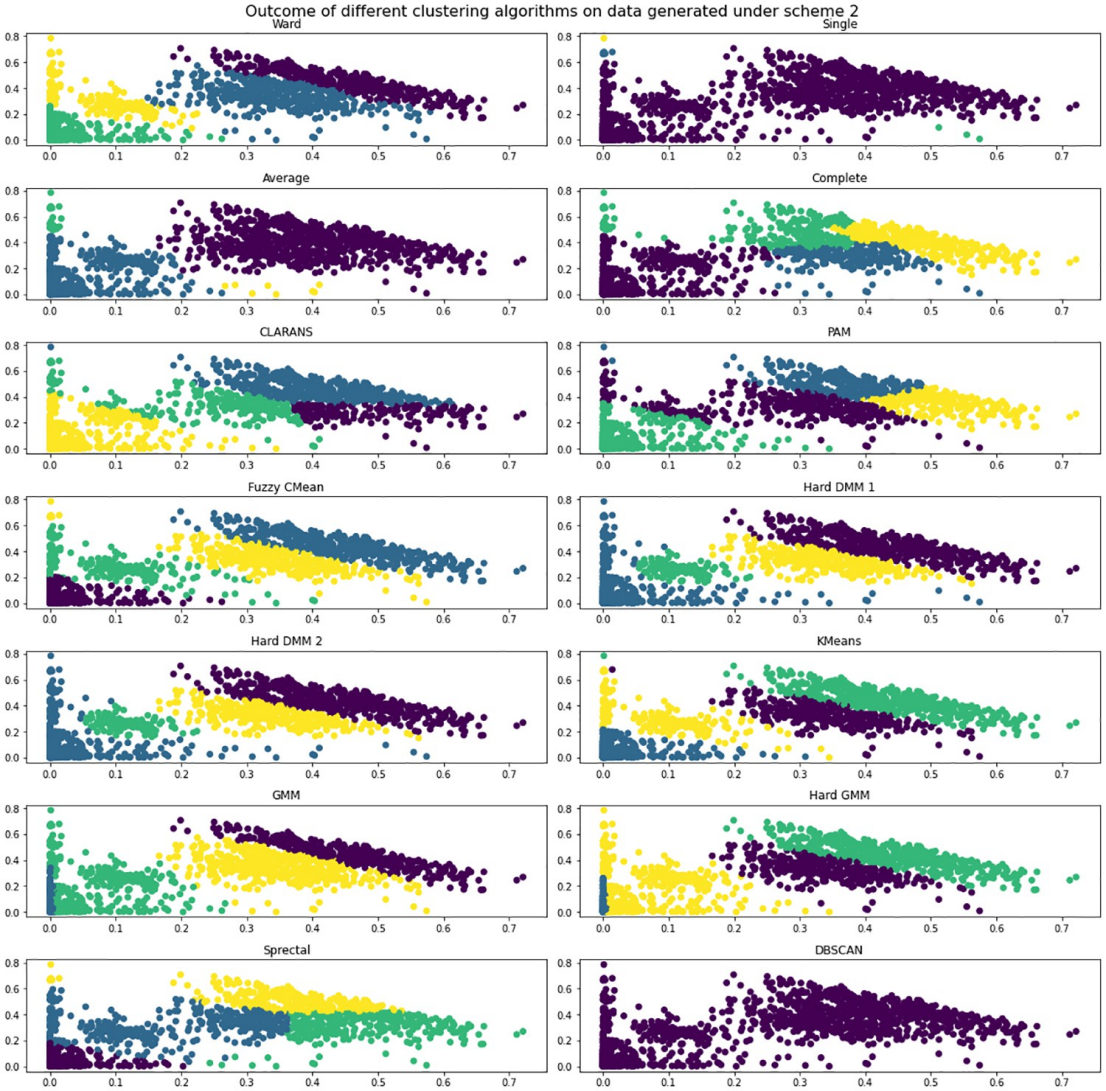
Outcome of different clustering algorithms on simulated data generated under scheme 1.

We have generated data under two schemes mentioned above and used different clustering algorithms to find patterns. We have measured the performance of algorithms in terms of accuracy, precision and recall. Fig 1 shows the data generated under scheme 1 with true clusters. And Fig 2 shows how different algorithms finds pattern on the data. We see that Hard DMM 1, Hard DMM 2, KMeans, GMM and Hard GMM recognize pattern in the data somewhat similar to the original pattern. Other algorithms fail to recognize the true patterns. The detailed result can be seen in Table 1. The data generated under scheme 2 is shown in Fig 3. We can see that the data has more complex patterns than data under scheme 1. From Fig 4, we see that only Hard DMM 1 and Hard DMM 2 are able to find pattern similar to the true patterns. All other algorithms fail to understand the true patterns of the generated data. The corresponding results in detail can be seen in Table 2.

**Table 1:** Accuracy, precision and recall of different clustering algorithms on data generated under scheme 1.

Methods	Accuracy	Precision	Recall
Hard DMM 1	**0.928889**	**0.897778**	**0.917724**
Hard DMM 2	**0.928889**	**0.897778**	**0.917724**
Ward	0.882222	0.877333	0.883416
Single	0.557778	0.337778	0.852264
Average	0.844444	0.854667	0.818948
Complete	0.711111	0.627111	0.615798
CLARANS	0.824444	0.833778	0.767597
PAM	0.805556	0.834889	0.741326
Fuzzy CMean	0.832222	0.854889	0.767149
KMeans	0.852222	0.869556	0.787949
GMM	0.882222	0.891111	0.891111
Hard GMM	0.921111	0.890444	0.910820
Sprectal	0.616667	0.664667	0.664667
DBSCAN	0.555556	0.333333	0.333333

**Table 2:** Accuracy, precision and recall of different clustering algorithms on data generated under scheme 2.

Methods	Accuracy	Precision	Recall
Hard DMM 1	**0.925385**	**0.928167**	**0.909739**
Hard DMM 2	**0.925385**	**0.928167**	**0.909739**
Ward	0.830000	0.858625	0.803385
Single	0.385385	0.250625	0.346824
Average	0.464615	0.495000	0.455818
Complete	0.490000	0.581208	0.511178
CLARANS	0.502308	0.531250	0.454768
PAM	0.510769	0.495250	0.512096
Fuzzy CMean	0.846923	0.877417	0.807752
KMeans	0.858462	0.882708	0.816642
GMM	0.726154	0.786875	0.786875
Hard GMM	0.756154	0.800833	0.764774
Sprectal	0.562308	0.632167	0.632167
DBSCAN	0.384615	0.250000	0.250000

**Fig 5:**
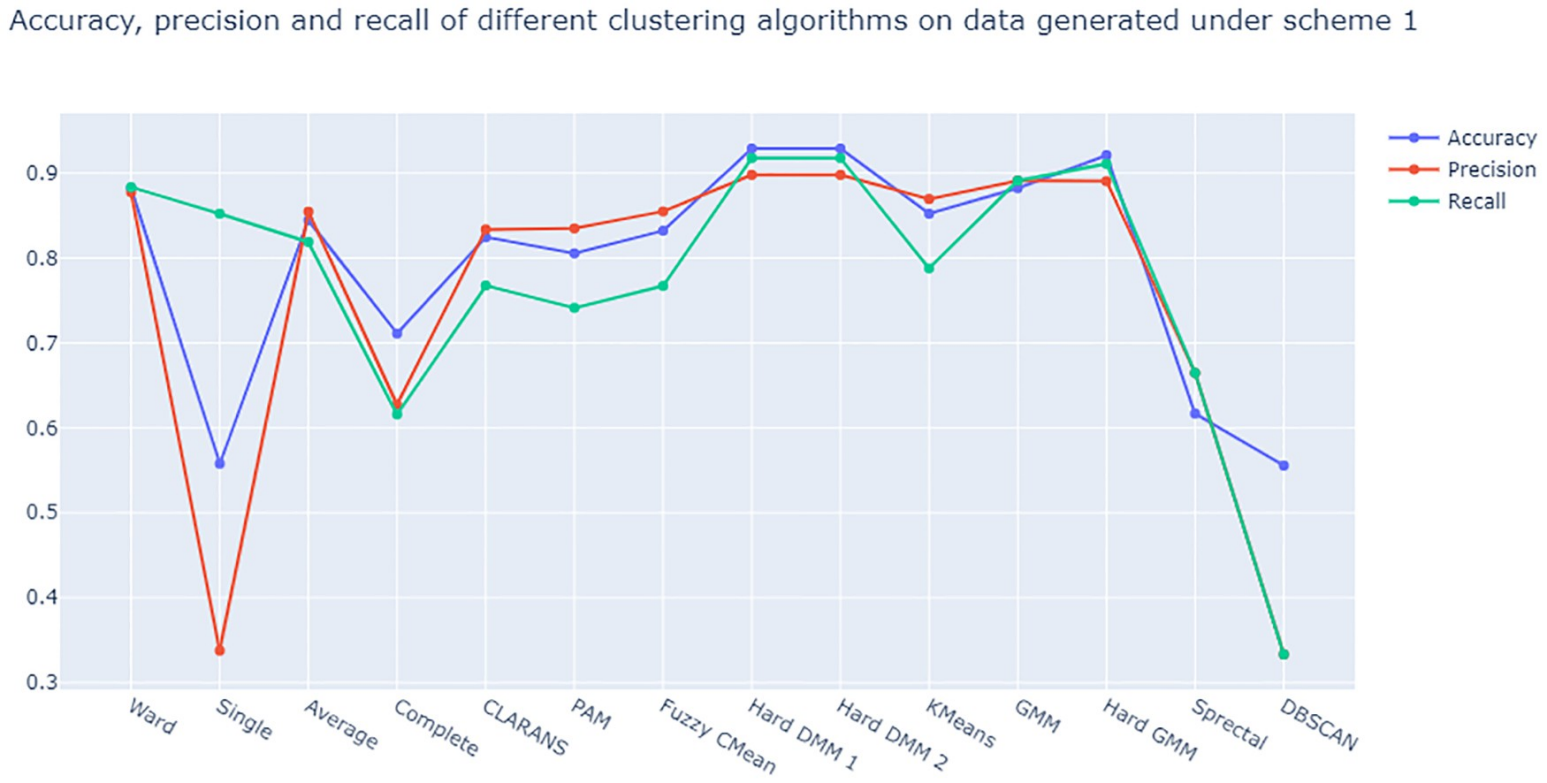
Plot of accuracy, precision and recall of different clustering algorithms on data generated under scheme 1.

**Fig 6:**
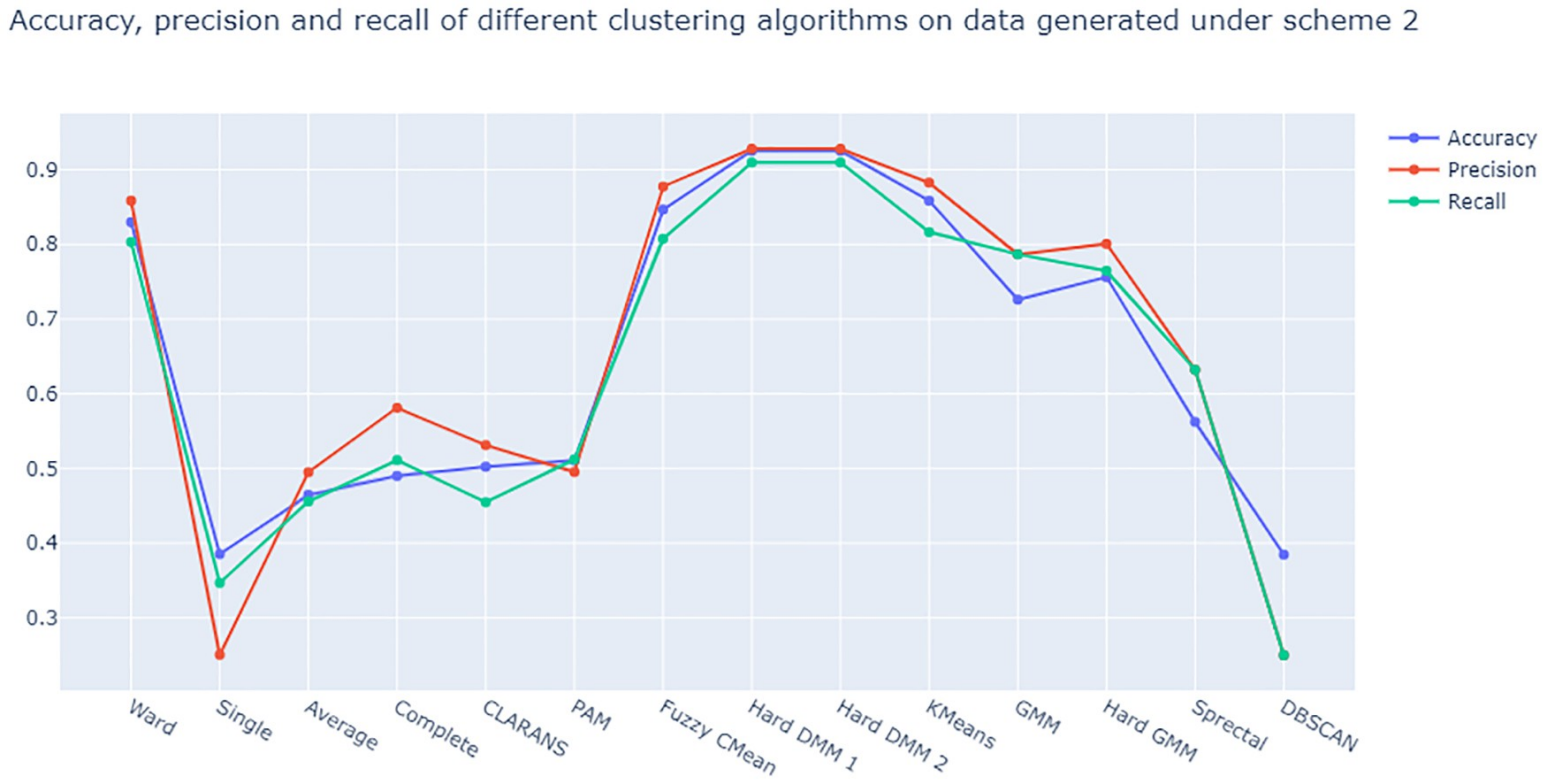
Plot of accuracy, precision and recall of different clustering algorithms on data generated under scheme 2.

From Fig 5 we see that Hard DMM 1 and Hard DMM 2 have the highest accuracy, precision and recall on data generated under scheme 1. On the other hand from Fig 6 also, we see that Hard DMM 1 and 2 work better than all other algorithms we considered when it comes to the data generated under scheme 2. Hard DMM 1 and Hard DMM 2 have shown best performance in terms of accuracy, precision and recall on our generated dataset. On this note, it is important to understand the interpretation of precision and recall in classification. Precision gives the ratio of number of elements correctly classified under a certain class (or cluster) and all number of elements which actually belong to that class (or cluster). On the other hand, recall gives the ratio of number of elements correctly classified under a certain class (or cluster) and total number of elements classified under that class both correctly and incorrectly. Even though both of these measures are very important, unfortunately we can not maximize both at the same time. When we have different algorithms with same accuracy, we must choose the model with better precision and recall. In our simulation study,both versions of Hard DMM have the best accuracy on both the dataset along with very good precision and recall. Scheme 2 produces a dataset with a complex, non spherical patterns which makes it very difficult to cluster with generic algorithms or mixture model with Gaussian distributions. When compositional data shows asymmetric and non spherical patterns, mixture model using Dirichlet distribution is expected to give better results as Dirichlet distribution can adopt both symmetric and asymmetric shapes. Our simulation study confirms that Hard DMM can be a suitable choice for both spherical and non spherical data when it comes to clustering.

### Performance testing

We wanted to test the performance of our proposed model (Hard DMM 1) for varied dimensions, varied number of clusters and varied overlap. We have simulated data with dimensions 5, 10, 20, 35, 50, 70 and 100 with number of clusters 2 to 6. For each dimension and number of clusters, we have generated data 100 times and used our proposed model to check accuracy. For clusters 2 to 5, we have set very less to none overlap in the data. And for 6 clusters we have introduced overlap in the data with increasing dimension. Let us recall that *p* denotes the dimension, *k* denotes the number of cluster and *α*_*j*_ = (*α*_*j*1_, …, *α*_*jp*_) denotes the parameters of Dirichlet distribution from mixture component *j*. The data generating schemes are mentioned below.

- ***k* = 2**: 800 random samples from a Dirichlet distribution, with *p* parameters drawn randomly from a range 1 and 110, sorted in ascending order. 500 random samples from a Dirichlet distribution, with *p* parameters drawn randomly from a range 1 and 110, sorted in descending order.
- ***k* = 3**: 500 random samples from a Dirichlet distribution, with *p* parameters drawn randomly from a range 110 and 500, sorted in ascending order. 400 random samples from a Dirichlet distribution, with *p* parameters drawn randomly from a range 1 and 110, sorted in descending order. 300 random samples from a Dirichlet distribution, with all *p* parameters equal to 50.
- ***k* = 4**: 400 random samples from a Dirichlet distribution, with *p* parameters drawn randomly from a range 1 and 100, sorted in ascending order. 300 random samples from a Dirichlet distribution, with *p* parameters drawn randomly from a range 1 and 100, sorted in descending order. 800 random samples from a Dirichlet distribution, with all *p* parameters equal to 50. 500 random samples from a Dirichlet distribution with *l* parameters equals to 110 and rest *p* − *l* parameters drawn randomly from Uniform (1,5) distribution, sorted in ascending order. *l* = (2, 3, 5, 8, 12, 15, 18) for *p* = (5, 10, 20, 35, 50, 70, 100) respectively.
- ***k* = 5**: 500 random samples from a Dirichlet distribution, with *p* parameters drawn randomly from a range 110 and 500, sorted in ascending order. 100 random samples from a Dirichlet distribution, with *p* parameters drawn randomly from a range 110 and 500, sorted in descending order. 300 random samples from a Dirichlet distribution, with *p* parameters drawn randomly from a range 1 and 110, sorted in ascending order. 400 random samples from a Dirichlet distribution, with *p* parameters drawn randomly from a range 1 and 110, sorted in descending order. 300 random samples from a Dirichlet distribution, with all *p* parameters equal to 50.
- ***k* = 6**: 500 random samples from a Dirichlet distribution, with *p* parameters drawn randomly from a range 110 and 500, sorted in ascending order. 100 random samples from a Dirichlet distribution, with *p* parameters drawn randomly from a range 110 and 500, sorted in descending order. 300 random samples from a Dirichlet distribution, with *p* parameters drawn randomly from a range 1 and 110, sorted in ascending order. 400 random samples from a Dirichlet distribution, with *p* parameters drawn randomly from a range 1 and 110, sorted in descending order. 300 random samples from a Dirichlet distribution, with all *p* parameters equal to 50. 500 random samples from a Dirichlet distribution with *l* parameters equals to 110 and rest *p* − *l* parameters drawn randomly from Uniform (1,5) distribution, sorted in ascending order. *l* = (2, 3, 5, 8, 12, 15, 18) for *p* = (5, 10, 20, 35, 50, 70, 100) respectively.

**Fig 7:**
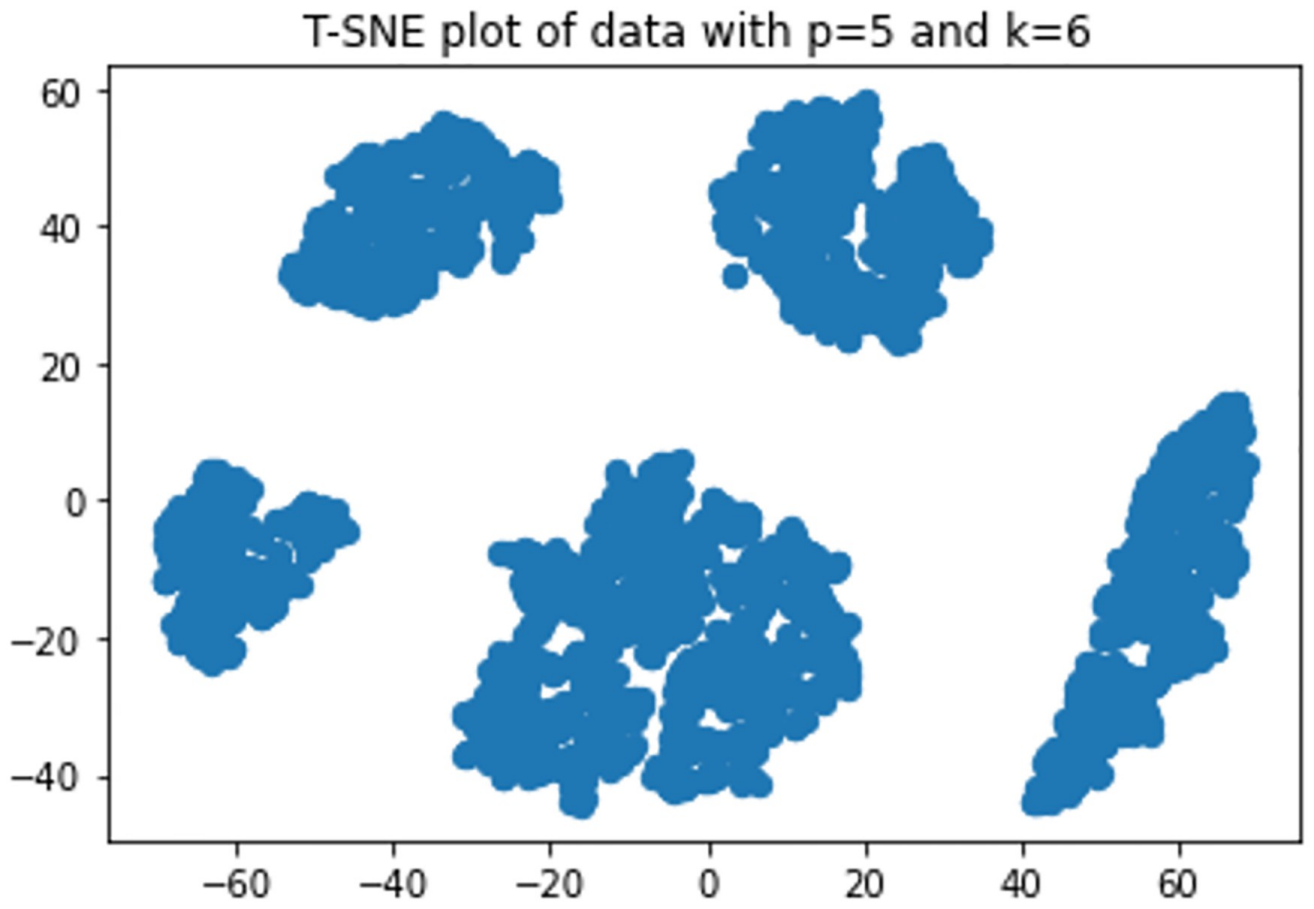
T-SNE plot of data with p = 5 and k = 6.

**Fig 8:**
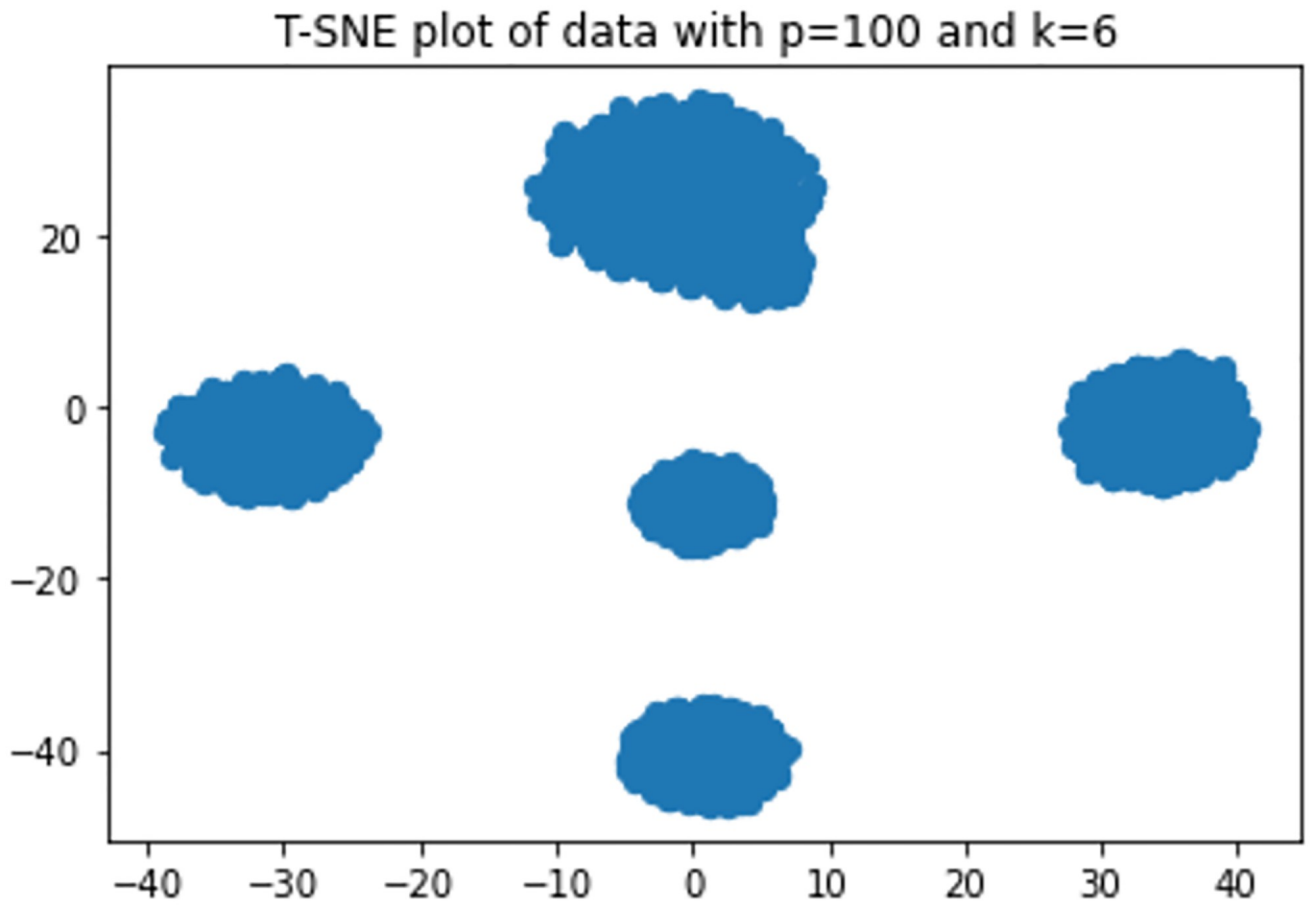
T-SNE plot of data with p = 100 and k = 6.

**Fig 9:**
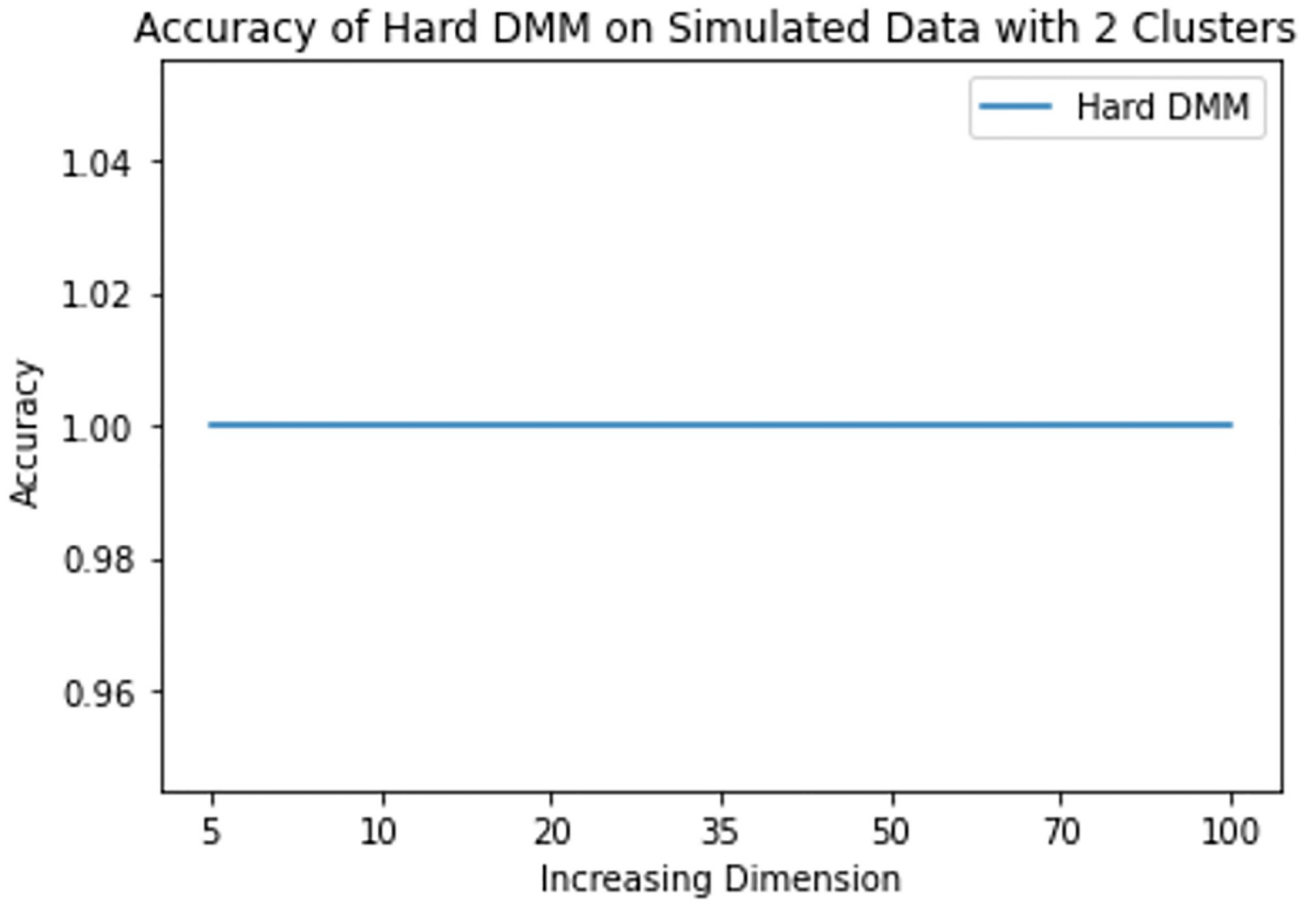
Mean accuracy of hard DMM 1 with 2 clusters and varied dimensions.

**Fig 10:**
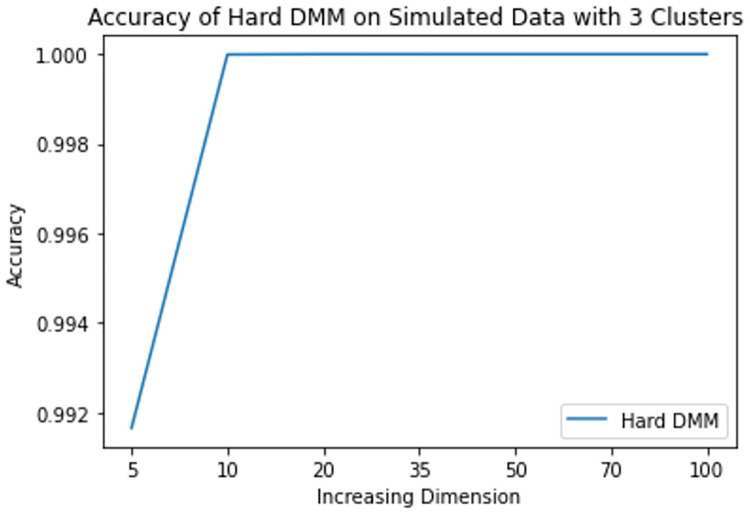
Mean accuracy of hard DMM 1 with 3 clusters and varied dimensions.

**Fig 11:**
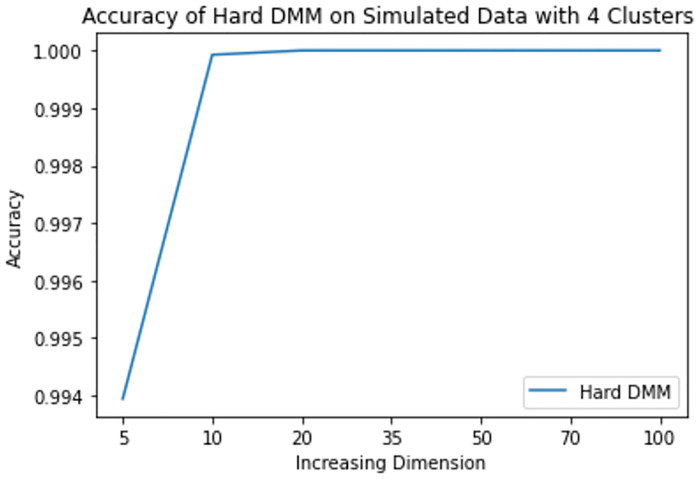
Mean accuracy of hard DMM 1 with 4 clusters and varied dimensions.

**Fig 12:**
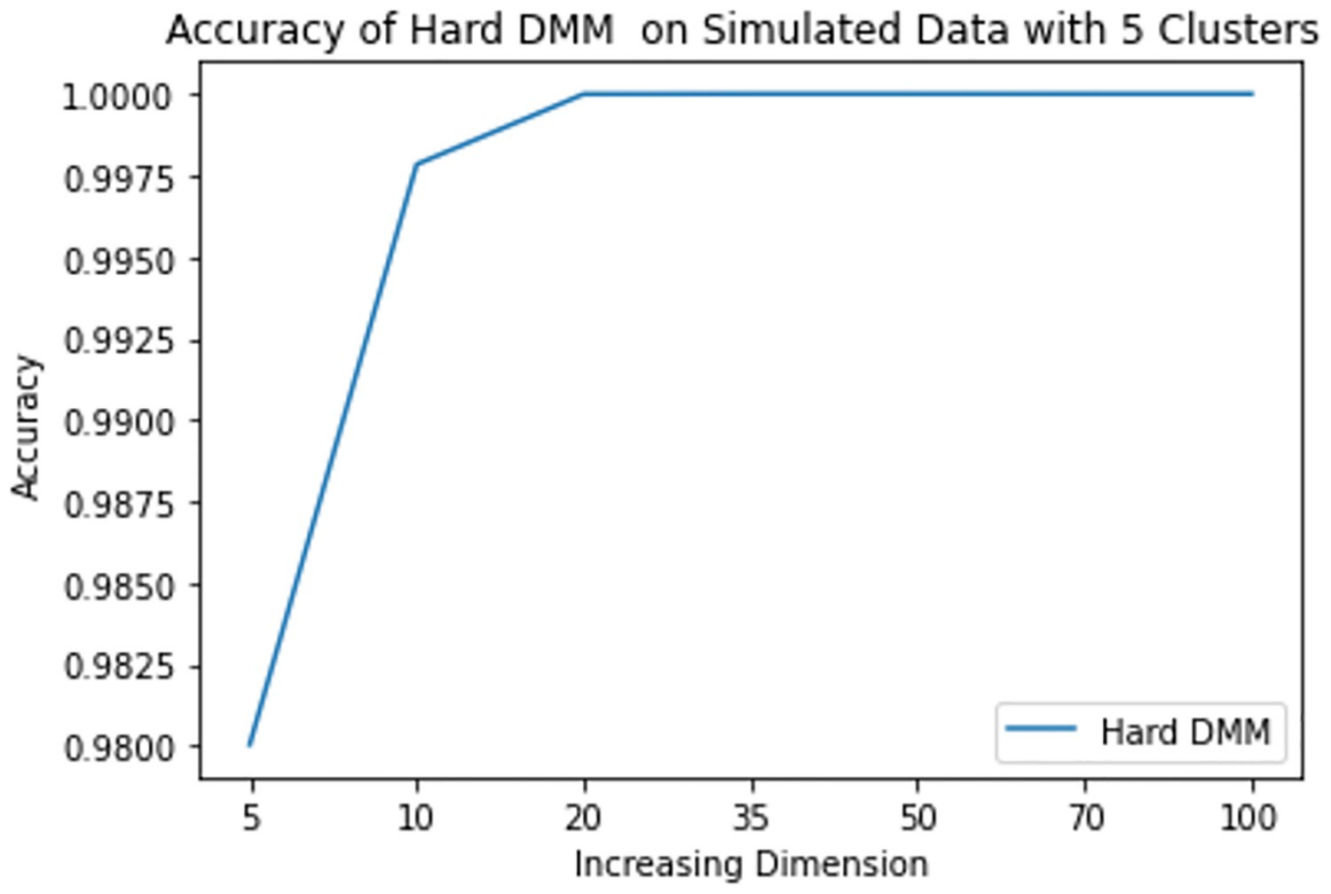
Mean accuracy of hard DMM 1 with 5 clusters and varied dimensions.

**Fig 13:**
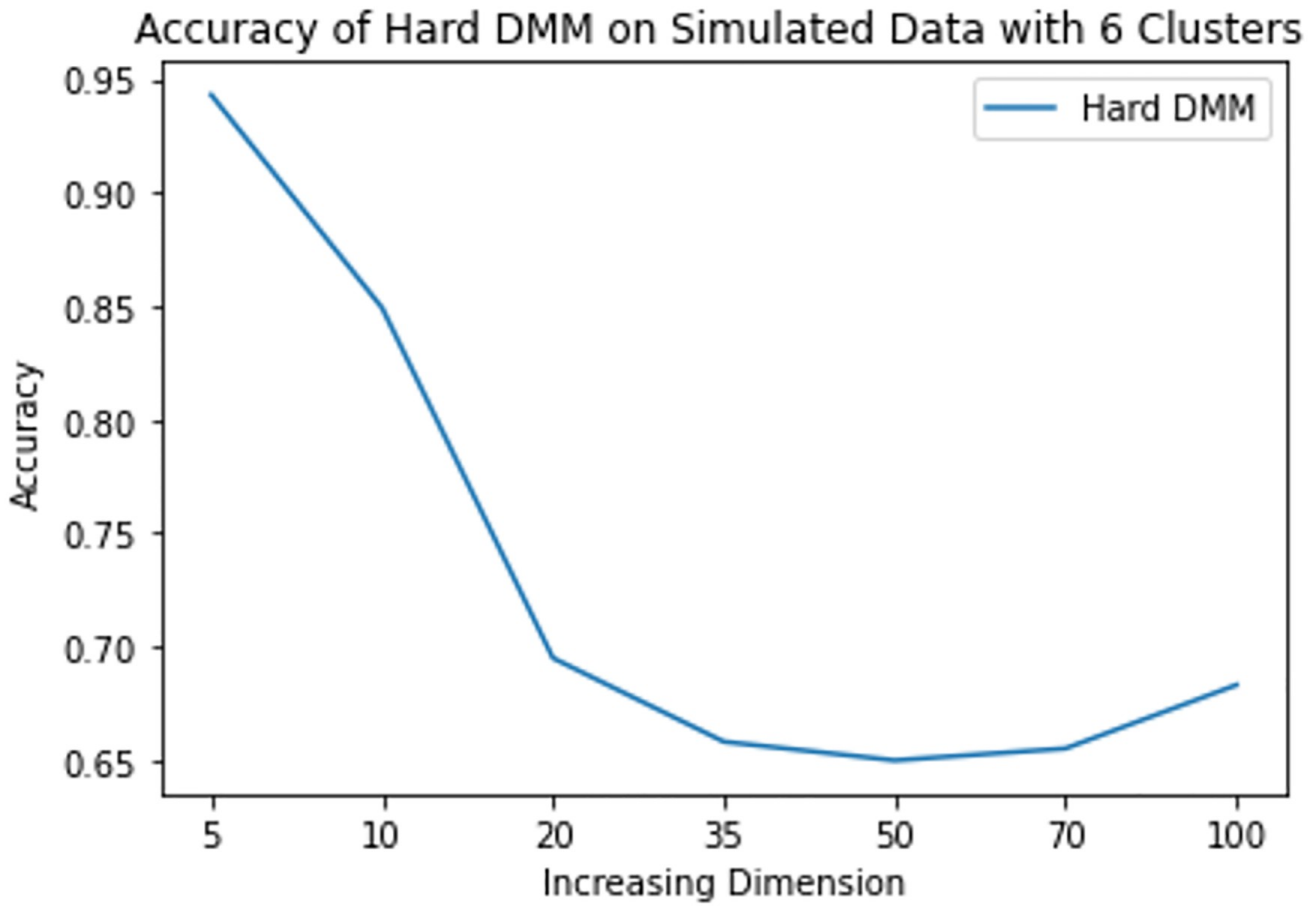
Mean accuracy of hard DMM 1 with 6 clusters, varied dimensions and increasing overlap.

The T-SNE [53, 54] plots in Figs 7 and 8 show that with *p* = 5 there is some overlap in one cluster and with *p* = 100 one cluster has completely been overlapped on another. The performances of the model for varied dimension, number of clusters and overlap is shown in Figs 9–13 respectively.

From results in Table 3, we see that increasing dimension and increasing number of clusters do not have much impact on the accuracy of Hard DMM. But increasing overlap has significant impact on the accuracy of Hard DMM. It is to be noted that many algorithms suffer from “Curse of Dimensionality” with increasing dimension in the data. For example, in case of GMM, a *p* dimensional mean vector and *p* × *p* symmetric covariance matrix need to be estimated for each *k* clusters. In other words for a GMM with *k* clusters and *p* dimensions, (*k* − 1) + *kp*(1 + (*p*/2 + 1/2)) number of parameters need to be estimated. On the other hand, in case of a DMM with *k* clusters and *p* dimensions, we need only (*k* − 1) + *kp* parameters to estimate. So for 10 clusters and 100 dimensional data GMM estimates 51509 parameters, whereas DMM estimates only 1009 parameters on the same situation. So, DMM has an added advantage over GMM when it comes to high dimensionality. That is why we have noticed in our study that increasing dimension has very little to none impact on the performance of Hard DMM. Also with increasing number of cluster, the number of parameters increase linearly. And with a good starting value in the EM algorithm, the model converges soon with satisfactory results. On the contrary, overlap in the data leads to misclassification, which in turn decreases the performance of Hard DMM significantly.

**Table 3:** Mean accuracy of hard DMM 1 with varied dimensions and number of clusters.

k	p = 5	p = 10	p = 20	p = 35	p = 50	p = 70	p = 100
2	1.000000	1.000000	1.000000	1.00000	1.00000	1.000000	1.000000
3	0.991658	0.999992	1.000000	1.00000	1.00000	1.000000	1.000000
4	0.993945	0.999925	1.000000	1.00000	1.00000	1.000000	1.000000
5	0.980044	0.997838	0.999994	1.00000	1.00000	1.000000	1.000000
6	0.942976	0.849214	0.695095	0.65829	0.65009	0.655305	0.683252

## Real data applications

We have applied the proposed methods on two real data problems. Our main idea was to check how our model works for the given data and not to provide an optimum solution for the problems. We have checked three measures to evaluate the performance, namely: accuracy, precision and recall. All measures have been compared with hierarchical agglomerative clustering with linkage criteria ward, single, average and complete, PAM, CLARANS, Fuzzy Cmean, KMeans GMM, Hard GMM, Spectral clustering and DBSCAN algorithm. At first we have checked for missing data. In our study there was no missing data. The class labels were subsequently label encoded in order to make it compatible with python. Using L1 normalization [55] the data was then converted into compositional data and zero values were treated using multiplicative replacement which is available in scikit-bio [56], a python library.

### Wholesale customers data

We have used a data from Marketing and Management domain for our first experiment. [57] has used this data for logical discriminant models. The dataset can be downloaded from UCI Machine Learning Repository. The data refers to 440 customers of a wholesale distributor, where 298 customers are from the Horeca (Hotel/Restaurant/Café) channel and the rest 142 customers are from the Retail channel. The wholesale customers are grouped in above two classes according to frequency spending degrees of four types:

- low frequency-low spending;
- high frequency-low spending;
- regular frequency-regular spending;
- high frequency-high spending.

**Fig 14:**
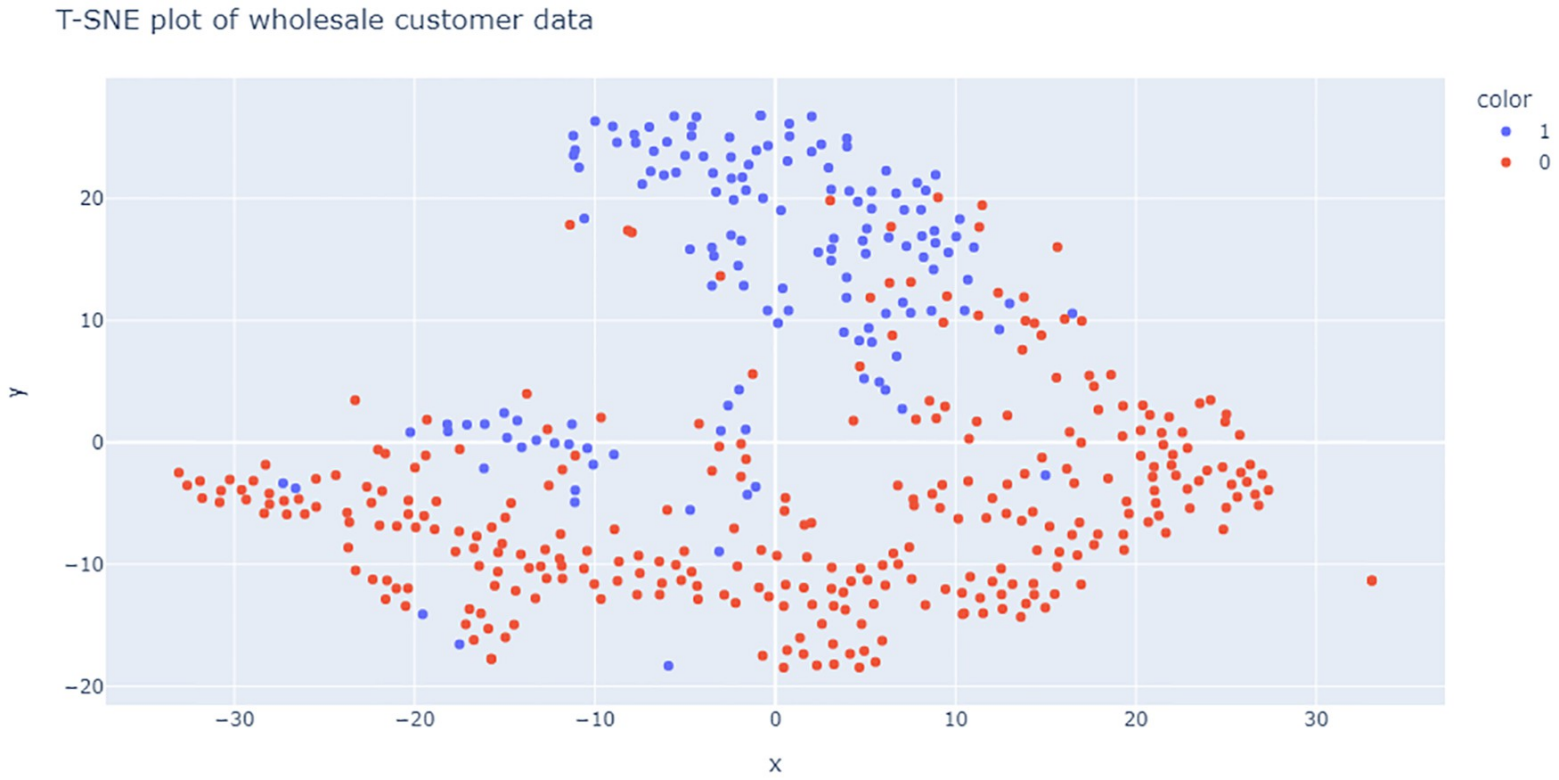
T-SNE plot of wholesale customer data.

The first two spending patterns are captured by the class Horeca and the second two patterns are captured by the class Retail. The wholesale data concerning the customers consists of annual spending in monetary units (m.u.) on different product categories, namely: fresh products, milk products, grocery, frozen products, detergents and paper products and delicatessen. The summary statistics of the data is shown in Table 4. The aim of the analysis is to find if there are different spending patterns for the two groups of customers and if so, maybe the wholesale could differentiate its marketing actions directed to these groups. For our study we will restrict ourselves to clustering analysis. In this case we have *p* = 6, *k* = 2 and *N* = 440. The T-SNE plots in Fig 14 shows complex distributional patterns. We have used different algorithms for clustering and checked their performances. The corresponding results are shown in Table 5.

**Table 4:** Summary statistics of wholesale customers data.

	Channel	Region	Fresh	Milk	Grocery	Frozen	Detergents_Paper	Delicassen
count	440.000	440.000	440.000	440.000	440.000	440.000	440.000	440.000
mean	1.322	2.543	12000.297	5796.265	7951.277	3071.931	2881.493	1524.870
std	0.468	0.774	12647.328	7380.377	9503.162	4854.673	4767.854	2820.105
min	1.000	1.000	3.000	55.000	3.000	25.000	3.000	3.000
25%	1.000	2.000	3127.750	1533.000	2153.000	742.250	256.750	408.250
50%	1.000	3.000	8504.000	3627.000	4755.500	1526.000	816.500	965.500
75%	2.000	3.000	16933.750	7190.250	10655.750	3554.250	3922.000	1820.250
max	2.000	3.000	112151.000	73498.000	92780.000	60869.000	40827.000	47943.000

**Table 5:** Performance comparison of different model based clustering methods on wholesale customers data.

Methods	Accuracy	Precision	Recall
Hard DMM 1	**0.770455**	0.780769	0.750448
Hard DMM 2	**0.770455**	0.780769	0.750448
Ward	0.756818	0.757798	0.732705
Single	0.675000	0.498322	0.338269
Average	0.768182	0.764344	0.741682
Complete	0.406818	0.320612	0.319944
CLARANS	0.718182	0.744021	0.713506
PAM	0.740909	0.755270	0.725436
Fuzzy CMean	0.731818	0.748558	0.718779
KMeans	0.731818	0.748558	0.718779
GMM	0.740909	**0.790292**	**0.790292**
Hard GMM	0.754545	0.789300	0.752955
Sprectal	0.734091	0.750236	0.750236
DBSCAN	0.677273	0.500000	0.500000

**Fig 15:**
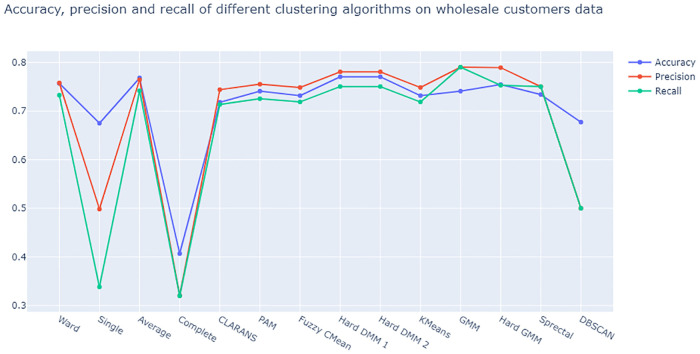
Plot of accuracy, precision and recall of different algorithms on on wholesale customers data.

From Fig 15 we see that both versions of Hard DMM work better than all other algorithms under consideration in terms of Accuracy. Precision and recall are found to be little better in GMM and Hard GMM than Hard DMM. As discussed before, when there are different algorithms with same level of accuracy, it is better to choose the algorithm with more precision and recall. In this experiment Hard DMM has the highest accuracy with comparatively good precision and recall value. So, Hard DMM can still be considered as a suitable choice in this situation.

### Wine data

**Fig 16:**
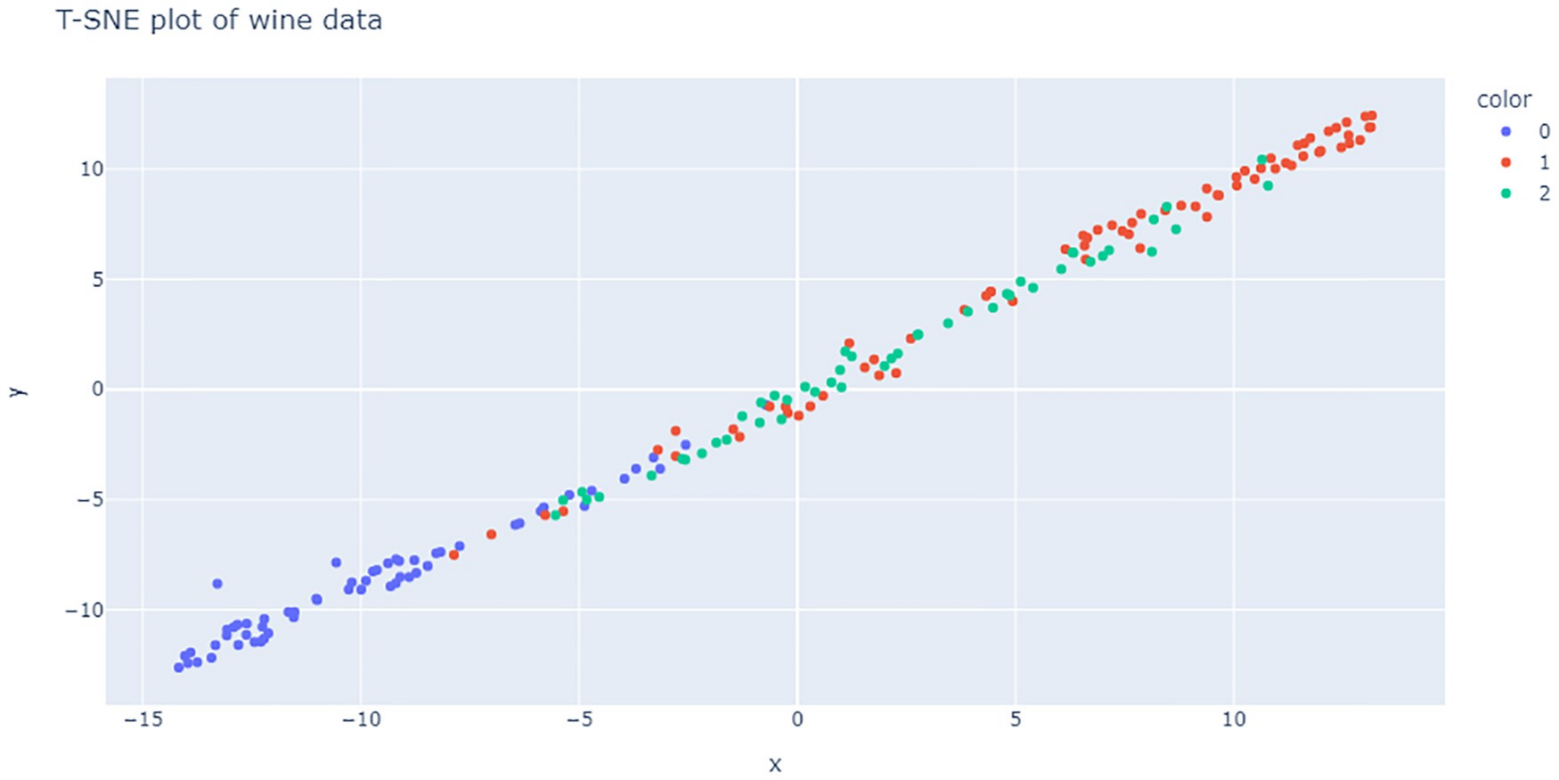
T-SNE plot of wine data.

The dataset we heve chosen for our second experiment is from physical science domain. These data are the results of a chemical analysis of wines grown in the same region in Italy but derived from three different cultivars. The analysis determined the quantities of 13 constituents found in each of the three types of wines. The 13 components are namely: Alcohol, Malic acid, Ash, Alcalinity of ash, Magnesium, Total phenols, Flavanoids, Nonflavanoid phenol,Proanthocyanins, Color intensity, Hue, OD280/OD315 of diluted wines and Proline. This data has been used by many researchers, (see [58–60]). This dataset can also be downloaded from UCI Machine Learning Repository. The aim of the analysis is to identify the different types of wines based on its components. But the attributes color intensity and hue do not constitute chemical components of wine. Hence, we have dropped those two variables for our compositional data analysis. We have used it for clustering purpose. In this case, we have *p* = 11, *k* = 3 and *N* = 178. The summary statistics of the data is shown in Table 6. Fig 16 displays the T-SNE plot of the data which shows difficult cluster patterns. Like before, we have performed clustering with different clustering algorithms. The results in detail is shown in Table 7.

**Table 6:** Summary statistics of wine data.

	0	1	2	3	4	5	6	7	8	9	10	11
count	178.000	178.0000	178.000	178.000	178.000	178.000	178.000	178.000	178.000	178.000	178.000	178.000
mean	1.938	13.000	2.336	2.366	19.494	99.741	2.295	2.029	0.361	1.590	2.611	746.893
std	0.775	0.811	1.117	0.274	3.339	14.282	0.625	0.998	0.124	0.572	0.709	314.907
min	1.000	11.030	0.740	1.360	10.600	70.000	0.980	0.340	0.130	0.410	1.270	278.000
25%	1.000	12.362	1.602	2.210	17.200	88.000	1.742	1.205	0.270	1.250	1.937	500.500
50%	2.000	13.050	1.865	2.360	19.500	98.000	2.355	2.135	0.340	1.555	2.780	673.500
75%	3.000	13.677	3.082	2.557	21.500	107.000	2.800	2.875	0.437	1.950	3.170	985.000
max	3.000	14.830	5.800	3.230	30.000	162.000	3.880	5.080	0.660	3.580	4.000	1680.000

**Table 7:** Performance comparison of different model based clustering methods on wine data.

Methods	Accuracy	Precision	Recall
Hard DMM 1	**0.674157**	**0.693130**	**0.749177**
Hard DMM 2	**0.674157**	**0.693130**	**0.749177**
Ward	0.646067	0.642660	0.689409
Single	0.398876	0.389671	0.427611
Average	0.584270	0.532527	0.521509
Complete	0.516854	0.490513	0.404811
CLARANS	0.612360	0.597660	0.591908
PAM	0.668539	0.672008	0.692386
Fuzzy CMean	0.511236	0.487177	0.490715
KMeans	0.511236	0.487177	0.486869
GMM	0.528090	0.494173	0.494173
Hard GMM	0.539326	0.521899	0.545030
Sprectal	0.612360	0.594180	0.594180
DBSCAN	0.331461	0.333333	0.333333

**Fig 17:**
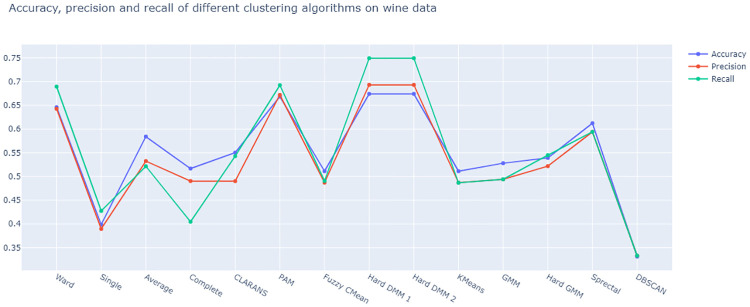
Plot of accuracy, precision and recall of different algorithms on on wine data.

From Fig 17, we see that, both versions of Hard DMM work better than all other clustering algorithms in terms of accuracy, precision and recall. For complex distributional pattern of compositional data, Hard DMM work better than other models as, compositional data can be naturally modelled using Dirichlet distribution.

## Conclusion

In this paper we have shown a convenient way to build Dirichlet mixture model to cluster compositional data. The model can be used without any transformation. In this case, Dirichlet distribution is a natural choice as it works with the unit sum constraint. Researchers generally use generic algorithms for clustering compositional data, whereas Hard DMM offers an exclusive solution specially for compositional data considering both the spherical and non spherical cluster patterns. Dirichlet distribution is well known for modelling symmetric and asymmetric data. This advantage can be exploited using Hard DMM.

We wanted to use a distribution other than normal in the mixture model and check whether it works as par with predominantly used methods such as GMM and KMeans. From the simulation study and two real data problems we see that when there is a pattern in the composition (proportions), both versions of Hard DMM are able to identify the clusters with quite a satisfactory result. For clustering purpose we had to be cautious while using data used for classification, as not always classification and clustering done on same ground. For example, if images are classified based on presence of a dog in it and the data contains values of red, green and blue channel, clustering algorithm tries to find completely a different pattern.

We have also done an extensive simulation study to evaluate the performance of our proposed method. We see that increasing number of dimensions (upto 100) and increasing number of clusters do not seem to have much effect on the performance. But increasing overlap makes the accuracy decrease accordingly. DMM can also be advantageous in high dimensional setting as it requires relatively less number of parameters to be estimated when compared to other mixture model. Due to the complexity of maximum likelihood (ML) estimation for Dirichlet parameters, Dirichlet distribution has long been ignored for clustering purpose. In our study, we have shown a novel way to use ML estimates of dirichlet parameters conveniently in mixture set up which can be used to cluster compositional data.

In our study, we have considered the number of clusters to be known in advance. But in reality, sometimes we need to estimate *k* before we can start clustering. We have also not explored the situation when the data is very high dimensional and sparse. Both the issues would require further research and we keep that for our future work.

Compositional data is getting very popular in biology domain. We hope to see more future applications of Dirichlet distribution and DMM in compositional data analysis.
